# Fluoroindate Glass Co-Doped with Yb^3+^/Ho^3+^ as a 2.85 μm Luminescent Source for MID-IR Sensing

**DOI:** 10.3390/s21062155

**Published:** 2021-03-19

**Authors:** Marcin Kochanowicz, Jacek Zmojda, Agata Baranowska, Marta Kuwik, Bartłomiej Starzyk, Magdalena Lesniak, Piotr Miluski, Wojciech A. Pisarski, Joanna Pisarska, Jan Dorosz, Maurizio Ferrari, Dominik Dorosz

**Affiliations:** 1 Department of Power Engineering, Photonics and Lighting Technology, Bialystok University of Technology, 45D Wiejska Street, 15-351 Bialystok, Poland; j.zmojda@pb.edu.pl (J.Z.); a.baranowska@pb.edu.pl (A.B.); p.miluski@pb.edu.pl (P.M.); doroszjan@pb.edu.pl (J.D.); 2 Institute of Chemistry, University of Silesia, 9 Szkolna Street, 40-007 Katowice, Poland; marta.kuwik@us.edu.pl (M.K.); joanna.pisarska@us.edu.pl (J.P.); 3 Faculty of Materials Science and Ceramics, AGH University of Science and Technology, 30 Mickiewicza Av., 30-059 Krakow, Poland; starzyk@agh.edu.pl (B.S.); mlesniak@agh.edu.pl (M.L.); ddorosz@agh.edu.pl (D.D.); 4 IFN-CNR CSMFO Lab. and FBK Photonics Unit, via alla Cascata 56/C Povo, 38123 Trento, Italy; maurizio.ferrari@ifn.cnr.it

**Keywords:** fluoroindate glass, luminescence MID-IR, CO_2_ sensing, Yb^3+^/Ho^3+^

## Abstract

This work reports on the fabrication and analysis of near-infrared and mid-infrared luminescence spectra and their decays in fluoroindate glasses co-doped with Yb^3+^/Ho^3+^. The attention has been paid to the analysis of the Yb^3+^ → Ho^3+^ energy transfer processed ions in fluoroindate glasses pumped by 976 nm laser diode. The most effective sensitization for 2 μm luminescence has been obtained in glass co-doped with 0.8YbF_3_/1.6HoF_3_. Further study in the mid-infrared spectral range (2.85 μm) showed that the maximum emission intensity has been obtained in fluoroindate glass co-doped with 0.1YbF_3_/1.4HoF_3_. The obtained efficiency of Yb^3+^ → Ho^3+^ energy transfer was calculated to be up to 61% (0.8YbF_3_/1.6HoF_3_), which confirms the possibility of obtaining an efficient glass or glass fiber infrared source for a MID-infrared (MID-IR) sensing application.

## 1. Introduction

The MID-infrared (MID-IR) optical sources operating in the 3 μm spectral range have attracted major attention due to their wide field of applications in the medical field, the military, and especially in remote sensing applications [1,2,3,4,5,6,7,8]. The MID-IR differential absorption lidar (DIAL) systems can be used for the construction of novel optical sensors of atmospheric chemistry. The Decadal Survey recommended missions to measure atmospheric constituents including CO_2_, CH_4_, CO, O_3_, NO_2_, SO_2_, and CH_2_O [9]. The atmospheric components noted above have unique and useful characteristic absorption features in the mid-infrared which enable their detection by using optical spectroscopy [10]. Moreover, ~3 μm lasers can be used as the pumping source of optical parametric oscillation (OPO) [11]. Two approaches have been widely investigated: bulk material lasers and fiber-based lasers [12]. Among low-phonon glasses, the fluoroindate glasses are very useful MID-IR photonic materials. Their properties like a wide transmission range (UV—10 μm), low phonon energy (c.a. 510 cm^−1^) acceptance of high concentration of rare-earth (RE) ions, and thermal stability which enables their fiberization, make them an attractive laser glass for optical fiber fabrication [13,14,15,16]. The main advantages of fluoroindate glasses with respect to the commonly known ZBLAN glasses are lower phonon energy, better thermal stability, and an extended IR absorption edge. Moreover, in comparison to the fluoro-zirconium and chalcogenide materials, it should be stated that fluoroindium glasses have better mechanical properties and resistance to chemical corrosion [17,18,19]. Besides, their nonlinear properties have been used in construction supercontinuum fiber sources operating up to 5.4 μm [20].

In the case of CO_2_ detection (2.7–3 μm absorption band), the Ho^3+^-doped fluoroindate glass (^5^I_6_ → ^5^I_7_ transition) is a promising candidate as a luminescent source of radiation and can be an alternative to semiconductor laser diodes [21]. Spectroscopic properties of the singly Ho^3+^-doped fluoroindate glasses in MID-IR spectral range have been reported before [22]. It should be noted that infrared luminescence studies of RE-co-doped fluoroindate glasses are practically limited to the Er^3+^/Tm^3+^ Er^3+^/Yb^3+^, Tm^3+^/Ho^3+^, and Er^3+^/Ho^3+^ [23,24,25,26].

It is well known that obtaining 2.85 μm efficient emissions from holmium is limited by two problems: the relatively short lifetime of the ^5^I_6_ level and the lack of suitable absorption bands matching with the current high power laser diode [27]. The first can be solved by using Pr^3+^ ions as a depopulator to quench the lower level of Ho^3+^: ^5^I_7_. The second problem can be solved through sensitization of the Ho^3+^ by Er^3+^ or Yb^3+^ ions and through obtaining luminescence by Yb^3+^, Er^3+^ → Ho^3+^ energy transfer [26,28,29,30,31].

In this paper, the effect of sensitization of holmium by Yb^3+^ ions on the near and mid-infrared emission properties is presented. Having in mind the practical application in MID-IR CO_2_ sensors, detailed analyses of the energy transfer mechanisms and RE-co-dopant optimization under commonly used 976 nm laser diode pumping have been performed. To the best of the authors’ knowledge, the emissions and energy transfer properties of the Yb^3+^/Ho^3+^ co-doped fluoroindate glasses have not been investigated before.

## 2. Materials and Methods

The investigated fluoroindate glasses have the following molar composition (38-x-y)InF_3_-20ZnF_2_-20SrF_2_-16BaF_2_-4GaF_3_-2LaF_3_-xYbF_3_–yHoF_3_, (x = 0.8; y = 0−1.6). The glass samples were prepared with high purity (99.99%) reagents. After homogenization of the components, 5 g glass bathes were placed in a covered platinum crucible. The 5 g glass sample is only enough for spectroscopic measurements. We are aware of the requirements for the fabrication of cylindrical or planar optical fibers (in bulk or fiber optics)—a larger number of glasses (20 g) are needed. Despite this, an exothermic (crystallization) peak at the 395 °C ΔT parameter (ΔT = Tx − Tg = 88 °C) confirmed the high thermal stability of the glass, which allows for using glasses in an optical fiber drawing process [26]. As someone may not agree with that stability parameter is an indicator for drawing (our team who deal with fiber drawing is not always familiar with this concept), it is known that fluoroindate glasses can be drawn into optical fibers [15,16]. Preparation, melting, and quenching have been done in a glove box (MBraun, Garching, Germany) in a nitrogen atmosphere (O_2_, H_2_O < 0.5 ppm). Labels and specified lanthanide co-dopant compositions have been included in Table 1.

In order to remove oxide impurities from the raw reagents and compensate for the loss of fluorine due to the formation and loss of HF in glass batch ammonium bifluoride (NH_4_HF_2_) was added as a fluorinating agent (14 wt.% higher amount of excess). The glass batches were firstly fluorinated at 270 °C for 2 h and then melted at 900 °C for 1 h. The melts were poured out onto a stainless teel plate and annealed at 290 °C for 2 h. Spectroscopic measurements in the wide range of 1000–3100 nm were carried out using an Acton 2300i monochromator equipped with a PbSe detector (Teledyne, Princeton Instruments, Acton, MA, USA) with a lock-in detection (Stanford Reseach Systems, Sunnyvale, CA, USA) setup and high power Roithner Lasertechnik GmbH (Vienna, Austria) laser diodes (λ_exc_ = 976 nm, P_opt(max)_=1 W). Infrared transmission spectrum was measured by using Fourier spectrometer Bruker Optics-Vertex70V (Billerica, MA, USA). Luminescence decay measurements were performed using a system PTI QuantaMaster QM40 coupled to a tunable pulsed optical parametric oscillator (OPO), pumped by the third harmonic of a Nd:YAG laser (OpotekOpolette 355 LD, Carlsbad, CA, USA). The laser system was equipped with a double 200 monochromator, a multimode UV-VIS photomultiplier tube (PMT) (R928), and Hamamatsu H10330B-75 detectors controlled by a computer. Luminescence decay curves were recorded and stored by a PTI ASOC-10 oscilloscope (Horiba, Northamption, UK). The accuracy of luminescence decay measurements was close to (±1 µs).

## 3. Results and Discussion

### 3.1. Infrared Transmission Spectrum and Hydroxide Group Content

**Figure 1:**
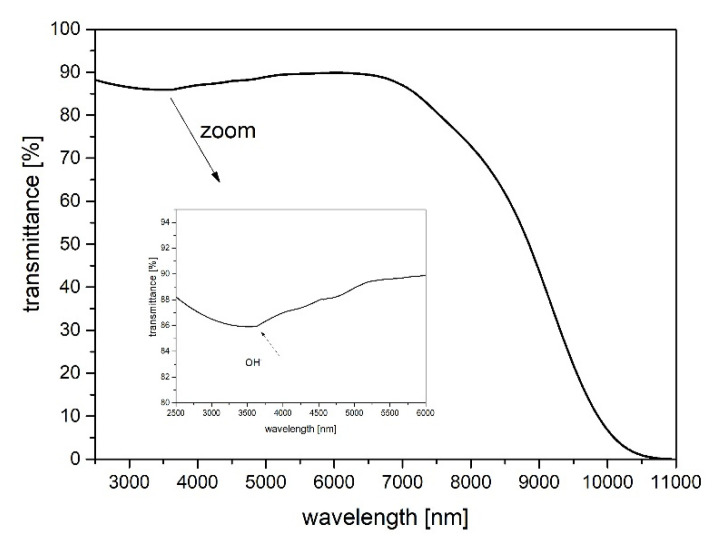
Transmittance spectrum of synthesized fluoroindate host glass in the mid-infrared range (thickness of the sample—2 mm).

The infrared transmittance spectrum of the prepared fluoroindate glass host (without rare-earth dopants) is presented in Figure 1. As shown, the IR transmission range is approximately up to 10 μm, which is much bigger than those of bismuth-germanate glass (5.5 μm) [32], bismuthate glass (5.5 μm) [33], ZBLAN glass (8 μm) [18], and fluoroaluminate glass (9 μm) [34].

However, regarding low-phonon glasses, only ZBLAN and fluoroindate can be drawn into high-quality optical fibers. Fluoroindate glass is one of the infrared materials that offers continuous optical transmission from the ultraviolet to midinfrared without any absorption peaks and can be drawn into good quality fibers, while their transmission window is much wider than standard zirconium fluoride based glass [35]. The absorption band at 3.3 μm shown in the inset of Figure 1 provides information about the hydroxide group content within the structure of each of the produced forms of glass. It is known that OH^−^ groups have a strong negative impact on MID-IR luminescence. The hydroxide concentration and the absorption coefficient at the wavelength of 3.1 µm and can be estimated according to the following equations [32,36]:

(1) $$\alpha_{OH^{-}}\left[cm^{-1}\right]=\frac{1}{d}ln\frac{T}{T_{b}}$$

(2) $$\alpha_{OH^{-}}\left[ppm\right]=\frac{1000}{d}log\frac{T}{T_{b}}$$

where: *d*—sample thickness, *T*—the value of the transmittance in the absorption peak, *T_b_*—the value of the transmittance at the baseline.

The calculated absorption coefficient and hydroxide groups content was found to be 0.088 cm^−1^ and 3.82 ppm, respectively. Since composition of the glasses host is very similar and they were prepared and melted in glove boxes, all glasses were found to have a similar amount of water. The obtained result is better than in fluorophosphate glass (35.5 ppm) [37] as well as fluoroaluminate glass (22.2 ppm) [34]. The reduced OH^−^ content results in an increase in quantum efficiency under the excitation of a 976 nm laser [11].

### 3.2. Luminescence Properties

**Figure 2:**
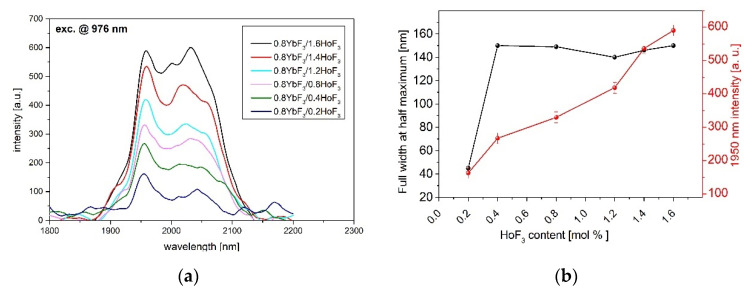
(**a**) Near- infrared (IR) luminescence spectra and (**b**) FWHM (Full With at Half Maximum), 1950 nm intensity of 0.8YbF_3_/(0.2–1.6)HoF_3_ co-doped fluoroindate glasses, λ_exc_ = 976 nm.

Measurements of the emission bands in the Visible (VIS), Near Infrared (NIR), as well as Mid Infrared (MID-IR), enable conducting a comprehensive analysis on the effect of acceptor (Ho^3+^) concentration on the luminescent properties. It is commonly known that the Ho^3+^ ion cannot be pumped by 976 nm. Therefore, holmium levels are populated through a Yb^3+^ → Ho^3+^ energy transfer process. Figure 2 presents 2 μm emissions (Ho^3+^: ^5^I_7_ → ^5^I_8_) of the fabricated fluoroindate glasses co-doped with a different molar concentration of HoF_3_ under a 976 nm laser excitation.

The linear increase of 2 μm luminescence intensity with the increasing of the HoF_3_ content was observed. A smaller distance between the donor and acceptor ions leads to the efficient energy transfer from the excited ^2^F_5/2_ energy level of ytterbium. It can be seen that the shape of the luminescence band slightly changes after increasing the Ho^3+^ content. It was presented in the literature that the four Stark emission bands, an equivalent model of four-level system for describing the 2 μm fluorescence band, can be used [38]. The four Stark emission bands centered at 1915, 1965, 2026, and 2078 nm can be distinguished. In the fabricated fluoroindate glasses, the I_2078nm_/I_1965nm_ intensity ratio increases upon increasing the Ho^3+^ content, which also influences the FWHM (Figure 2b) [38].

**Figure 3:**
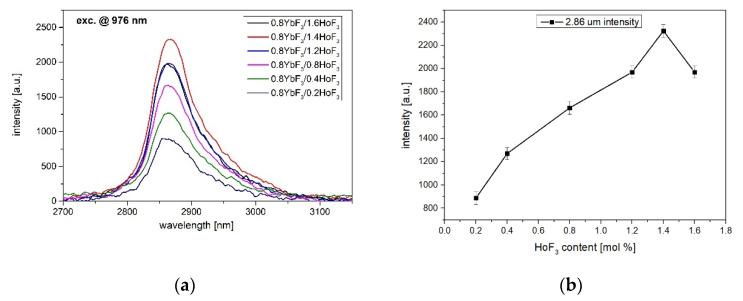
(**a**) Mid-IR luminescence spectra, (**b**) 2.86 μm emision intensity of 0.8YbF_3_/(0.2–1.6)HoF_3_ co-doped fluoroindate glasses, λ_exc_ = 976 nm.

Figure 3a shows the MID-IR emission spectra of 0.8YbF_3_/(0.2–1.6 mol%)HoF_3_ co-doped fluoroindate glasses excited at 976 nm. Due to the Ho^3+^: ^5^I_6_ → ^5^I_7_ transition, the broad and intense luminescence band at 2.85 μm was measured. It was also found that 2.85 μm luminescence intensity increases upon increasing Ho^3+^ ions up to 1.4HoF_3_ (0.8Yb-1.4Ho glass) and then reduces with further increasing of HoF_3_ (Figure 3b).

Table 2 shows that the obtained FWHM = 83 nm (Full With at Half Maximum) is only lower than in germanate glass, which indicates that fluoroindate glass is a promising low-honon energy glass for the construction of broadband MID-IR optical sources.

**Figure 4:**
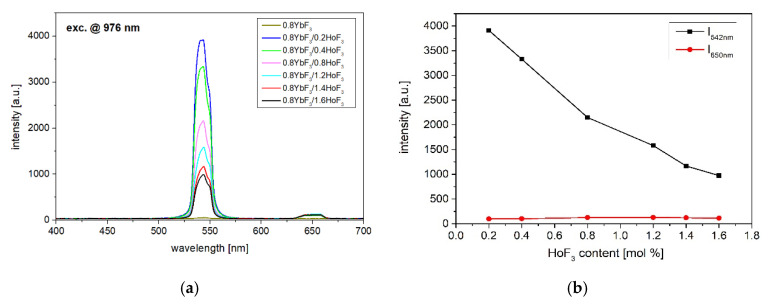
(**a**) Upconversion luminescence spectra and (**b**) intensity of upconversion emission bands of 0.8YbF_3_/(0.2–1.6)HoF_3_ co-doped fluoroindate glasses, λ_exc_ = 976 nm.

In the case of low phonon fluoroindate glasses co-doped with Yb^3+^/Ho^3+^ ions, the upconversion process cannot be neglected. Two emission bands at 542 nm and 650 nm corresponding to the ^5^F_4_,^5^S_2_ → ^5^I_8_ and ^5^F_5_ → ^5^I_8_ transitions of Ho^3+^ ions have been observed (Figure 4). It can be seen that the intensity of the green emission band reduces monotonically with the increasing of Ho^3+^ content. A similar effect has been observed in fluoroaluminate glass [34]. Reduced upconversion luminescence (reduced ESA2) allows us to get enhanced MID-IR emissions (Ho^3+^: ^5^I_6_ → ^5^I_8_). This was also confirmed by shortening the lifetime of the Ho^3+^: ^5^I_6_ level.

### 3.3. Energy Transfer Mechanism between Yb^3+^and Ho^3+^, Energy Transfer Efficiency

**Figure 5:**
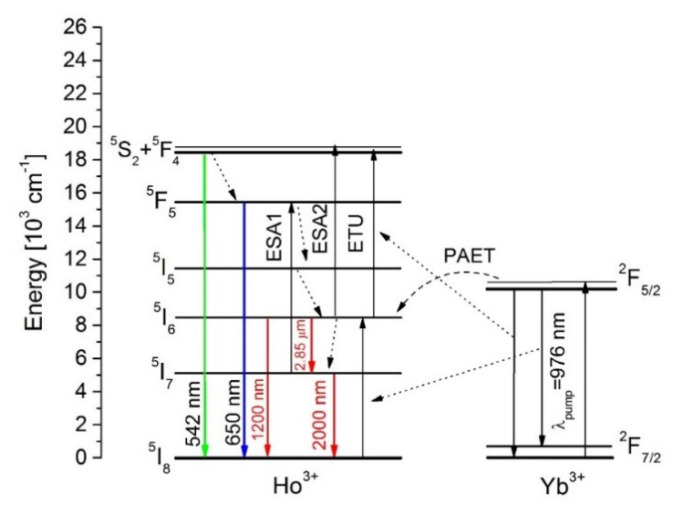
Simplified energy level diagram of Yb^3+^/Ho^3+^ co-doped fluoroindate glass. The energy transfer mechanisms under excitation at 976 nm are also indicated.

To explain the 2.85 μm, 2 μm, and upconversion emissions as well as the energy transfer mechanism in Yb^3+^/Ho^3+^ under 976 nm laser excitation, the energy level diagram was proposed based on the previous investigations and presented in Figure 5 [32,41].

Firstly, under 976 nm excitation, the Yb^3+^: ^2^F_5/2_ was populated directly through the ground state absorption process. Then, part of the energy was transferred to the Ho^3+^: ^5^I_6_ level by a phonon-assisted (PAET): Yb^3+^: ^2^F_5/2_ → Ho^3+^: ^5^I_6_ energy transfer. Part of Ho^3+^ ions could relax radiatively due to Ho^3+^: ^5^I_6_ → ^5^I_7_ (2.85 μm) and Ho^3+^: ^5^I_6_ → ^5^I_8_ (1200 nm). Another part of Ho^3+^ ions relaxed nonradiatively to the Ho^3+^: ^5^I_7_ level, which is responsible for 2 μm emissions (Ho^3+^:^5^I_7_ → ^5^I_8_). Due to the low phonon energy of the fluoroindate glasses (510 cm^−1^) upconversion mechanisms also had to be analyzed. Simultaneously, another excited Yb^3+^ ion populated the Ho^3+^: ^5^S_2_(^5^F_4_) level due to the energy transfer upconversion process (ETU). Some of the ions relaxed non-radiatively to the Ho^3+^: ^5^F_5_ level and the red emission (^5^F_5_ → ^5^I_8_) occurred. In addition, ions at Ho^3+^: ^5^I_6_ level were populated to the upper Ho^3+^: ^5^S_2_(^5^F_4_)_,_^5^F_5_ levels through excited-state absorption (ESA1 and ESA2). Finally, the green emission corresponding to the ^5^S_2_(^5^F_4_) → ^5^I_8_ transition took place [41].

**Figure 6:**
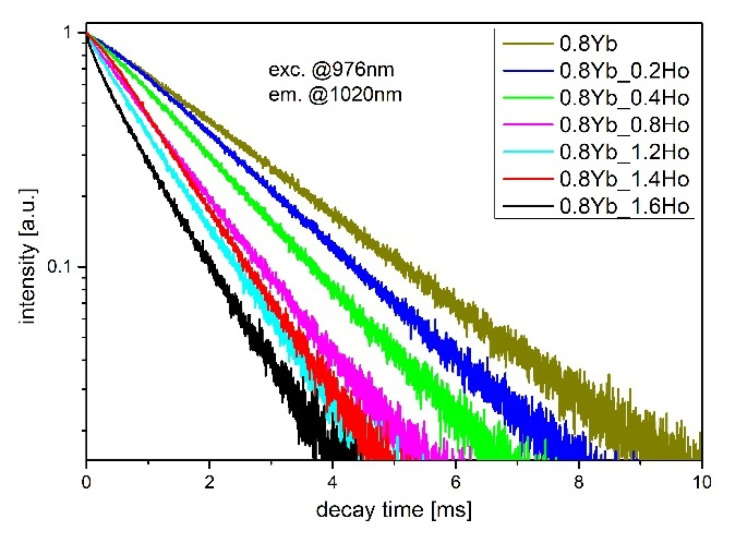
Luminescence decay curves from Yb^3+^: ^2^F_5/2_ level in Yb^3+^/Ho^3+^ co-doped fluoroindate glasses, λ_exc_ = 976 nm.

To determine the efficiency of energy transfer mechanisms, the luminescence decays in the fabricated glasses were analyzed. First of all, we focused on the depopulation process in the Yb^3+^: ^2^F_5/2_ level as a function of holmium ions. Shortening of the Yb^3+^: ^2^F_5/2_ lifetime occurred due to the Yb^3+^ → Ho^3+^ energy transfer. Figure 6 shows the luminescence decays of ^2^F_5/2_ level under 976 nm excitation. All measured luminescence decays were fitted by the single exponential curve. In singly Yb^3+^-doped fluoroindate glass, the measured lifetime is 2.25 ms and in Yb^3+^/Ho^3+^ co-doped glasses, the lifetime decreases with successive introduction of Ho^3+^ ions.

**Figure 7:**
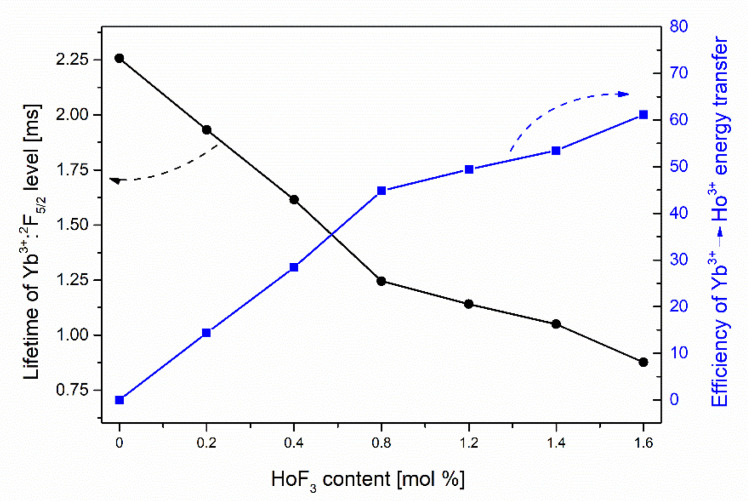
The Yb^3+^: ^2^F_5/2_ luminescence lifetime and the efficiency of the Yb^3+^ → Ho^3+^ energy transfer as a function of HoF_3_ content.

This effect confirms that Yb^3+^ is a suitable sensitizer in fabricated glasses and can efficiently transfer the energy to Ho^3+^ ions through a phonon-assisted process. According to ^2^F_5/2_ (Yb^3+^) lifetime changes, the efficiency of Yb^3+^ → Ho^3+^ energy transfer can be estimated by the following equation:

(3) $$\eta_{Yb\to Ho}=1-\frac{\tau_{Yb\left(Ho\right)}}{\tau_{Yb}}$$

where *τ_Yb_*_(_*_Ho_*_)_ and *τ_Yb_* are the lifetimes of ^2^F_5/2_ level in glasses co-doped with Yb^3+^/Ho^3+^ ions and singly doped with Yb^3+^ ions, respectively. In the fabricated glass, the maximum efficiency of ET was estimated to be close to 61% for the sample with 0.8YbF_3_/1.6HoF_3_ (Figure 7).

**Figure 8:**
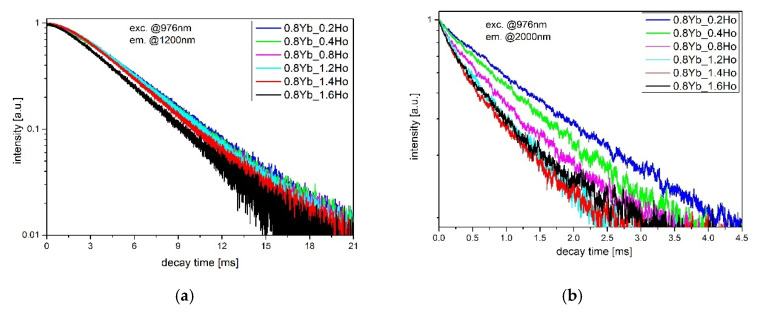
Luminescence decay curves from the Ho^3+^: ^5^I_6_ level (**a**) and ^5^I_7_ level (**b**) in Yb^3+^/Ho^3+^ co-doped fluoroindate glasses, λ_exc_ = 976 nm.

To analyze the overall distribution of excitation energy in Yb^3+^/Ho^3+^ ions, the luminescence decays of Ho^3+^: ^5^I_6_ and ^5^I_7_ levels were also measured (Figure 8). It can be seen that the lifetime of ^5^I_6_ level decreased slightly with an increasing Ho^3+^ concentration. This reduction leads to the limitation of ESA2 process [34], hence strong quenching in upconversion luminescence at 546 nm (Ho^3+^: ^5^S_2_ + ^5^F_4_) was observed (Figure 4). Simultaneously, the weak upconversion emission can promote the Ho^3+^: ^5^I_6_ → ^5^I_7_ transition, where the monotonical increase in 2.85 µm emission intensity occurs up to 1.4HoF_3_ co-doped glasses (Figure 3). The decay curves of the ^5^I_7_ energy level of the fluoroindate glass co-doped with Yb^3+^/Ho^3+^ ions were shown in Figure 8b. In this case, we can see that the addition of Ho^3+^ ions also reduces the lifetime at the wavelength of 2 µm (Ho^3+^:^5^I_7_ → ^5^I_8_). Based on the simplified energy diagram of Yb^3+^ and Ho^3+^ ions, we deduced that a shorter decay time on ^5^I_7_ level can restrain the ESA1 process and amplify the intensity of 2µm emission. Moreover, the faster radiative relaxation of ^5^I_7_ than ^5^I_6_ level supports inversion in the ions population between these levels, which is beneficial for the population accumulation of upper level of ~2.85 µm emissions, which originated from Ho^3+^: ^5^I_6_ → ^5^I_7_ transition [42].

In both analyzed cases, decay curves are not linear, hence we used a double-exponential function to estimate the average lifetime. In the case of double-exponential decay of luminescence, we used a known equation for calculating the average lifetime of higher energy levels in Ho^3+^ ions. The luminescence intensity could be described by the sum of two exponential decay components from:

(4) $$I\left(t\right)=A_{1}\exp\left(-\frac{t}{\tau_{1}}\right)+A_{2}\exp\left(-\frac{t}{\tau_{2}}\right)$$

where τ_1_ and τ_2_ were short- and long-decay components, respectively. Parameters A_1_ and A_2_ were fitting constants. Then, the average lifetime <τ> was given by:

(5) $$\langle \tau \rangle =\frac{A_{1}\tau_{1}^{2}+A_{2}\tau_{2}^{2}}{A_{1}\tau_{1}+A_{2}\tau_{2}}.$$

Calculated values of lifetimes of ^5^I_6_ and ^5^I_7_ levels are listed in Table 3.

The decrease in decay time with increasing Ho^3+^ concentration can be explained as the increased probability of the energy migration process between Ho-Ho pairs. A similar effect has been observed in barium gallo-germanate glasses [43].

## 4. Conclusions

Fluoroindate glasses co-doped with Yb^3+^/Ho^3+^ were fabricated and their spectroscopic properties under 976 nm laser diode excitation were characterized. The infrared transmission spectrum a indicates low OH^−^ content (single ppm level) in the fabricated fluoroindate glass. It confirms that the quenching of the MID-nfrared luminescence is strongly limited. In particular, the effect of Ho^3+^ sensitization by Yb^3+^ ions on luminescence spectra in the VIS, NIR, and MID-IR spectral ranges was analyzed. The maximum intensity of 2.85 μm emissions (Ho^3+^:^5^I_6_ → ^5^I_7_) has been obtained in glass co-doped with 0.8YbF_3_/1.4HoF_3_. Simultaneously, the reduction of the green emissions with increasing Ho^3+^ content was observed. This effect was a result of the reduced lifetime of the Ho^3+^:^5^I_6_ level (thus the ESA2 process). Finally, enhancement of the 2.85 μm luminescence took place. Analysis of the luminescence decay curves revealed that Ho^3+^ ions are efficiently sensibilizated. The maximum efficiency of Yb^3+^ to Ho^3+^ energy transfer was calculated to be up to 61%.

In summary, based on our results, the effective sensitization of Ho^3+^ in fluoroindate glasses for NIR and especially MID-IR emission can be realized by Yb^3+^. The fabricated glass can be used as a bulk glass or glass fiber luminescence source of 2.85 μm radiation for CO_2_ absorption-based sensing.

## Figures and Tables

**Table 1:** The molar percentage of holmium and ytterbium fluoride co-dopants.

Glass Sample	Co-Dopants	
	(x) YbF_3_ [mol%]	(y) HoF_3_ [mol%]
0.8Yb	0.8	0
0.8Yb-0.2Ho	0.8	0.2
0.8Yb-0.4Ho	0.8	0.4
0.8Yb-0.8Ho	0.8	0.8
0.8Yb-1.2Ho	0.8	1.2
0.8Yb-1.4Ho	0.8	1.4
0.8Yb-1.6Ho	0.8	1.6

**Table 2:** Comparison of the FWHM of MID-IR emission band in glasses and crystals doped with Ho^3+^.

Material	Dopants	FWHM	Ref.
LuLiF_4_ crystal	1.58 × 10^20^ Ho^3+^ (ions/cm^3^)	<50 nm	[39]
Chalcogenide glass	1Ho_2_S_3_ (wt.%)	43 nm	[40]
Germanate glass	1Yb_2_O_3_/0.17Ho_2_O_3_ (mol%)	110 nm	[36]
Bismuth-germanate glass	0.75Yb_2_O_3_/0.25Ho_2_O_3_ (mol%)	71 nm	[32]
Fluoroaluminate glass	2YbF_3_/0.5HoF_3_ (mol%)	59 nm	[34]
Fluoroindate glass	0.8YbF_3_/1.4HoF_3_ (mol%)	83 nm	This work

**Table 3:** Calculated lifetimes of ^5^I_6_ and ^5^I_7_ levels in Ho^3+^ ions.

Glass Code	Lifetime @1200 nm [ms]	Lifetime @2000 nm [ms]
0.8Yb_0.2Ho	5.13	1.69
0.8Yb_0.4Ho	5.01	1.41
0.8Yb_0.8Ho	4.84	1.10
0.8Yb_1.2Ho	4.98	0.89
0.8Yb_1.4Ho	4.71	0.77
0.8Yb_1.6Ho	4.27	0.88

## Data Availability

The data presented in this study are available on request from the corresponding Author.
